# Relationship Between Major Depression Symptom Severity and Sleep Collected Using a Wristband Wearable Device: Multicenter Longitudinal Observational Study

**DOI:** 10.2196/24604

**Published:** 2021-04-12

**Authors:** Yuezhou Zhang, Amos A Folarin, Shaoxiong Sun, Nicholas Cummins, Rebecca Bendayan, Yatharth Ranjan, Zulqarnain Rashid, Pauline Conde, Callum Stewart, Petroula Laiou, Faith Matcham, Katie M White, Femke Lamers, Sara Siddi, Sara Simblett, Inez Myin-Germeys, Aki Rintala, Til Wykes, Josep Maria Haro, Brenda WJH Penninx, Vaibhav A Narayan, Matthew Hotopf, Richard JB Dobson, RADAR-CNS Consortium

**Affiliations:** 1 Department of Biostatistics & Health Informatics Institute of Psychiatry, Psychology and Neuroscience King's College London London United Kingdom; 2 Institute of Health Informatics University College London London United Kingdom; 3 South London and Maudsley National Health Services Foundation Trust London United Kingdom; 4 Department of Psychological Medicine Institute of Psychiatry, Psychology and Neuroscience King's College London London United Kingdom; 5 Department of Psychiatry Amsterdam Public Health Research Institute and Amsterdam Neuroscience Amsterdam University Medical Centre, Vrije Universiteit and GGZ inGeest Amsterdam Netherlands; 6 Teaching Research and Innovation Unit Parc Sanitari Sant Joan de Déu, Fundació Sant Joan de Déu Barcelona Spain; 7 Centro de Investigación Biomédica en Red de Salud Mental Madrid Spain; 8 Faculty of Medicine and Health Sciences Universitat de Barcelona Barcelona Spain; 9 Department of Psychology Institute of Psychiatry, Psychology and Neuroscience King's College London London United Kingdom; 10 Center for Contextual Psychiatry Department of Neurosciences Katholieke Universiteit Leuven Leuven Belgium; 11 Faculty of Social Services and Health Care LAB University of Applied Sciences Lahti Finland; 12 Janssen Research and Development LLC Titusville, NJ United States; 13 see Acknowledgments

**Keywords:** mobile health (mHealth), mental health, depression, sleep, wearable device, monitoring

## Abstract

**Background:**

Sleep problems tend to vary according to the course of the disorder in individuals with mental health problems. Research in mental health has associated sleep pathologies with depression. However, the gold standard for sleep assessment, polysomnography (PSG), is not suitable for long-term, continuous monitoring of daily sleep, and methods such as sleep diaries rely on subjective recall, which is qualitative and inaccurate. Wearable devices, on the other hand, provide a low-cost and convenient means to monitor sleep in home settings.

**Objective:**

The main aim of this study was to devise and extract sleep features from data collected using a wearable device and analyze their associations with depressive symptom severity and sleep quality as measured by the self-assessed Patient Health Questionnaire 8-item (PHQ-8).

**Methods:**

Daily sleep data were collected passively by Fitbit wristband devices, and depressive symptom severity was self-reported every 2 weeks by the PHQ-8. The data used in this paper included 2812 PHQ-8 records from 368 participants recruited from 3 study sites in the Netherlands, Spain, and the United Kingdom. We extracted 18 sleep features from Fitbit data that describe participant sleep in the following 5 aspects: sleep architecture, sleep stability, sleep quality, insomnia, and hypersomnia. Linear mixed regression models were used to explore associations between sleep features and depressive symptom severity. The *z* score was used to evaluate the significance of the coefficient of each feature.

**Results:**

We tested our models on the entire dataset and separately on the data of 3 different study sites. We identified 14 sleep features that were significantly (*P*<.05) associated with the PHQ-8 score on the entire dataset, among them awake time percentage (*z*=5.45, *P*<.001), awakening times (z=5.53, *P*<.001), insomnia (z=4.55, *P*<.001), mean sleep offset time (z=6.19, *P*<.001), and hypersomnia (z=5.30, *P*<.001) were the top 5 features ranked by *z* score statistics. Associations between sleep features and PHQ-8 scores varied across different sites, possibly due to differences in the populations. We observed that many of our findings were consistent with previous studies, which used other measurements to assess sleep, such as PSG and sleep questionnaires.

**Conclusions:**

We demonstrated that several derived sleep features extracted from consumer wearable devices show potential for the remote measurement of sleep as biomarkers of depression in real-world settings. These findings may provide the basis for the development of clinical tools to passively monitor disease state and trajectory, with minimal burden on the participant.

## Introduction

According to the report of the World Health Organization, the total number of people with depression was estimated to exceed 300 million in 2015, equivalent to 4.4% of the world’s population [1]. There are several depression-related adverse outcomes, including premature mortality [2], decline in quality of life [3], and loss of occupational function [4].

Sleep disturbances are prevalent among depression patients; more than 90% of patients with depression reported poor sleep quality [5]. Sleep disturbances cover a wide range of different symptoms and disorders including insomnia, hypersomnia, excessive daytime sleepiness, and circadian rhythm disturbance [6]. Insomnia and sleep quality have been observed to be bidirectionally related to depression in several longitudinal studies [6]. Hypersomnia is more frequently present in depressive episodes of bipolar patients [7,8]. Changes in sleep architecture, such as reduced deep sleep, increased rapid eye movement (REM) sleep, and shortened REM latency, are also significant predictors of depression [9,10].

The gold standard for sleep evaluation is polysomnography (PSG), which involves several physiological measurements including electroencephalogram, electrocardiogram, electromyogram, and accelerometers [11]. Using PSG to assess sleep lacks ecological validity and is time-consuming, expensive, and labor-intensive, requiring dedicated equipment and separate laboratory rooms as well as experts to analyze the physiological signals. Since depression can affect patients for an extended period, long-term monitoring of sleep quality is essential. Due to the above shortcomings, PSG is not suitable for long-term sleep monitoring [12]. A sleep questionnaire, such as the Pittsburgh Sleep Quality Index (PSQI) [13], is another useful method to assess sleep. This method relies on the self-reporting of subjective factors, like low recall of sleep, that may affect the accuracy of the assessment [14].

Several recent studies have used wearable devices to estimate sleep quality and sleep-related parameters [15-18] and analyzed the relationship between sleep and depression [19-21]. Miwa et al [19] estimated sleep quality by detecting rollover movements during sleep and observed a significant difference in sleep quality between nondepressed and depressed people. Mark et al [20] estimated the sleep duration of 40 information workers for 12 days using a Fitbit wristband and found that sleep duration was positively correlated with mood. DeMasi et al [21] found that sleep was significantly related to changes in depressive symptoms. These studies have mostly been performed on single center and relatively small datasets (number of participants fewer than 100). Moreover, most of these studies only used basic sleep parameters, such as sleep duration; detailed information on sleep architecture, sleep patterns, and stability of sleep was not considered. The relationship between detailed sleep features, as estimated from data supplied by wearable devices, and depression is yet to be fully explored.

The first aim of this study was to design more sleep-related features, from wearable device data, that reflect the sleep architecture, sleep stability, sleep quality, and sleep disturbances (insomnia and hypersomnia) of the participant. The second aim was to explore associations between these sleep features and depressive symptom severity on a relatively large, multisite dataset. The third aim was to compare our findings with previous studies that used other measurements to assess sleep such as PSG and sleep questionnaires.

## Methods

### Dataset

#### Study Participants and Settings

The data we used in this paper were collected from a major EU Innovative Medicines Initiative research project, Remote Assessment of Disease and Relapse–Central Nervous System (RADAR-CNS) [22]. This project aims to investigate the use of remote measurement technologies to monitor people with depression, epilepsy, and multiple sclerosis in real-world settings. The study protocol for the depression component (Remote Assessment of Disease and Relapse–Major Depressive Disorder [RADAR-MDD]) is described in detail in Matcham et al [23]. The RADAR-MDD project aims to recruit 600 participants with a recent history of depression in 3 study sites (King’s College London [KCL], UK; Vrije Universiteit Medisch Centrum [VUMC], Amsterdam, The Netherlands; and Centro de Investigación Biomédican en Red [CIBER], Barcelona, Spain). Recruitment procedures vary slightly across sites and eligible participants are identified either through existing research cohorts (in KCL and VUmc) who had given consent to be contacted for research purposes; advertisements in general practices, psychologist practices, newspapers, and Hersenonderzoek.nl [24], which is a Dutch online registry (VUmc); or through mental health services (in KCL and CIBER) [23]. Participants from KCL and VUmc are community-based, while the participants from CIBER come from a clinical population. As part of the study, participants are asked to install several remote monitoring technology apps and use an activity tracker for up to 2 years of follow-up. Many categories of passive and active data are being collected and uploaded to an open-source platform, RADAR-base [25]. In this paper, we focus on the sleep and Patient Health Questionnaire 8-item (PHQ-8) data [26].

#### Sleep Data

According to the American Academy of Sleep Medicine manual for the scoring of sleep and associated events, sleep can be divided into 2 phases, REM sleep and non-REM (NREM) sleep, and NREM sleep can be subdivided into N1, N2, and N3 stages according to characteristic patterns of brain waves collected by PSG [11]. In our project, the daily sleep records of participants were collected by the Charge 2 or Charge 3 (Fitbit Inc). An entire night’s sleep is divided into 4 stages: awake, light, deep, and REM. The light stage provides estimates for the N1 and N2 stages in PSG, while the deep stage provides estimates for the N3 stage in PSG. According to several validation studies of Fitbit, the Fitbit wristband had limited specificity in sleep stages estimation [27-29]. Therefore, in this study, we were not expecting the Fitbit devices to provide information as accurate as PSG would have provided. However, the Fitbit devices were deemed sensitive enough to detect changes in sleep-wake states [27-29]; therefore, the provided sleep stage information could be used to determine estimates for detailed sleep parameters based on known sleep pathology.

#### PHQ-8 Data

The variability of each participant’s depressive symptom severity was measured via the PHQ-8, conducted by mobile phone every 2 weeks. The questionnaire contains 8 questions, with the score of each subitem ranging from 0 to 3. The total score (range 0 to 24) of all subitems is the PHQ-8 score, which can evaluate depressive symptom severity of the participant for the past 2 weeks. A PHQ-8 score ≥10 is the most commonly recommended cutpoint for clinically significant depressive symptoms [26] (ie, if the PHQ-8 of a participant is ≥10, the participant is likely to have had depressive symptoms in the previous 2 weeks). In the PHQ-8, subitem 3 refers to sleep. The content of subitem 3 is “Trouble falling or staying asleep, or sleeping too much” [26]. A higher score in subitem 3 indicates worse self-reported sleep in the past 2 weeks. For reading convenience, we denoted the score of subitem 3 as the sleep subscore in this paper.

#### Sociodemographics

Sociodemographic of participants were collected during the enrollment session. According to previous studies on the associations between depression and sociodemographic characteristics [30,31], we considered baseline age, gender, education level, and annual income as potential confounding variables in our analyses. Due to the different educational systems in different countries, we simply divided the education level into 2 levels: degree (or above) and below degree. The annual income levels of Spain and the Netherlands were transformed into equivalent British levels.

### Feature Extraction

#### Feature Window Size

For each PHQ-8 record, we extracted sleep features from a 2-week time window prior to the PHQ-8 completion time, as the PHQ-8 score is used to represent the depressive symptom severity of the participant for the past 2 weeks. The feature window is denoted as ∆t in this paper.

#### Sleep Features

According to known sleep pathology and our experience, 18 sleep features extracted in this paper were divided into the following 5 categories (Table 1): sleep architecture, representing the basic and cyclical patterns of sleep; sleep stability, representing the variance of sleep in the feature window; sleep quality, measures relating to total sleep and wake times; insomnia, trouble falling or staying asleep; and hypersomnia, excessive sleepiness.

**Table 1:** A list of sleep features used in this study and their short descriptions.

Features		Description	Unit
**Sleep architecture**			
	Av_tst	Mean total sleep time	Hour
	Av_time_bed	Mean time in bed	Hour
	Deep_pct	Mean percentage of deep sleep	%
	Light_pct	Mean percentage of light sleep	%
	REM_pct	Mean percentage of REM^a^ sleep	%
	NREM_pct	Mean percentage of NREM^b^ sleep	%
	Awake_pct	Mean percentage of awake time	%
	Av_onset	Mean sleep onset time	Hour
	Av_offset	Mean sleep offset time	Hour
	REM_L	Mean REM latency time	Hour
**Sleep stability**			
	Std_tst	Standard deviation of total sleep time	Hour
	Std_onset	Standard deviation of sleep onset time	Hour
	Std_offset	Standard deviation of sleep offset time	Hour
**Sleep quality**			
	Efficiency	Mean sleep efficiency	%
	Awake_5	Mean number of awakenings (>5 minutes) per night	Times
	WKD_diff	Total sleep time difference between weekend and weekdays	Hour
**Insomnia**			
	M_insomnia	Percentage of days with potential middle insomnia	%
**Hypersomnia**			
	Dur_10	Percentage of days with total sleep time >10 hours	%

^a^REM: rapid eye movement.

^b^Non-REM: non–rapid eye movement.

##### Sleep Architecture

The features of sleep architecture were intended to describe the basic and cyclical patterns of sleep. Therefore, we extracted some features similar to those in the PSG report (total sleep time, time in bed, sleep onset time, sleep offset time, and REM latency) [32], and features of the percentages of all sleep stages. Total sleep time of one night is defined as the sum of all nonawake stages (light, deep, and REM) [32]. The mean total sleep time in ∆t was denoted as *Av_tst*. Time in bed of one night is defined as the sum of all sleep stages (awake, light, deep, and REM) of the entire night [32]. The mean time in bed in ∆t was denoted as *Av_time_bed*. Percentage of each sleep stage is defined as the percentage of the time in the sleep stage to the time in bed of the entire night. Different sleep stages have different functions and can reflect the quality of sleep. Deep sleep is considered essential for memory consolidation [33], and REM sleep favors the preservation of memory [34]. A previous sleep report has shown that more deep sleep and fewer awakenings represent better sleep quality [32]. Therefore, we extracted the mean percentages of these 4 sleep stages in ∆t, and denoted them as *Deep_pct*, *Light_pct*, *REM_pct*, *Awake_pct*, respectively. The combination of deep and light sleep is NREM sleep. The mental activity that occurs in NREM and REM sleep is a result of 2 different mind generators, which also explains the difference in mental activity [35]. So, we extracted the mean percentage of NREM sleep in ∆t, which was denoted as *NREM_pct*. We calculated the mean sleep onset time (the first nonawake stage) in ∆t, denoted as *Av_onset*. Mean sleep offset time (the last nonawake stage) in ∆t was calculated and denoted as *Av_offset*. Previous literature has shown that shortened REM latency can be considered as a biological mark of depression relapse [9]. REM latency is defined as the interval between sleep onset and occurrence of the first REM stage. The mean REM latency in ∆t was denoted as *REM_L*.

##### Sleep Stability

The features in this category were used to estimate the variance of sleep during ∆t. We extracted the standard deviation of total sleep time, sleep onset time, and sleep offset time in ∆t, which were denoted as *Std_tst*, *Std_onset*, and *Std_offset*, respectively.

##### Sleep Quality

In this paper, we used features of sleep efficiency, awakenings, and weekend catch-up sleep to describe sleep quality. The definition of sleep efficiency is the percentage of total sleep time to time in bed [32]. Mean sleep efficiency in ∆t was denoted as *Efficiency*. The definition of awakenings (>5 minutes) for one night is the number of episodes in which an individual is awake for more than 5 minutes [32]. The average number of awakenings in ∆t was denoted as *Awake_5*. Weekend catch-up sleep is an indicator of insufficient weekday sleep, which might be associated with depression level [36]. A longer total sleep time during the weekend compared with weekdays may reflect the actual sleep needed [37]. Therefore, we calculated the mean total sleep time difference between weekend and weekdays in ∆t, which was denoted as *WKD_diff*.

##### Insomnia

A review of several longitudinal studies suggested that insomnia is bidirectionally related to depression [6]. According to the diagnostic features provided in the *Diagnostic and Statistical Manual of Mental Disorders, Fifth Edition* [38], insomnia manifests as initial insomnia (difficulty initiating sleep at bedtime), middle insomnia (frequent or prolonged awakening throughout the night), and late insomnia (early-morning awakening with an inability to return to sleep).

For initial insomnia and late insomnia, mean sleep onset time (*Av_onset*) and sleep offset time (*Av_offset*) can be used to partially reflect them, respectively. We define potential middle insomnia to be whether the total sleep time is less than 6 hours and there is at least one prolonged awakening (≥30 minutes) during the night. The percentage of days with potential middle insomnia in the feature window was denoted as *M_Insomnia*.

##### Hypersomnia

Hypersomnia can be another symptom of depression [7]. The hypersomnia criteria used in Tam et al [39] is sleeping more than 10 hours per day, 3 days per week. In this paper, the percentage of days with total sleep time greater than 10 hours was extracted in ∆t and denoted as *Dur_10*.

### Statistical Method

#### Data Inclusion Criteria

Sleep and PHQ-8 records were missing in our data cohort for a variety of expected reasons, including the participants not wearing the Fitbit wristband when they slept, participants forgetting to complete the PHQ-8, and the Fitbit wristband being damaged during follow-up. We, therefore, specified the following inclusion criteria: (1) PHQ-8 record should be completed (ie, participant answered all 8 questions in the questionnaire); (2) number of days with sleep records in the feature window should be at least 12 days (approximately 85% of the feature window size) [40]; (3) number of PHQ-8 records for each participant should be greater than or equal to 3 [41]; (4) date of PHQ-8 records should be before February 2020, because the impact of the COVID-19 pandemic on sleep needs to be excluded [42].

#### Statistical Analyses

In our study, each participant had multiple PHQ-8 records and repeated sleep measures. For this reason, we used linear mixed models, which allow for accounting of both within and between-individual variability over time [43]. For each sleep feature, a 3-level linear mixed model with a participant-specific random intercept and a site-specific random intercept was built on the entire dataset to explore the association between this sleep feature and depressive symptom severity (PHQ-8) by bivariate analysis. We then used 2-level linear mixed models with participant-specific random intercepts to test these associations on the 3 subsets (KCL, CIBER, and VUmc) separately. We similarly analyzed the associations between sleep features and sleep subscore. All models were adjusted for baseline age, gender, education level, and annual income, which were specified as fixed effects. Model assumptions were checked by the histograms of residuals and Q-Q plots. If the residuals are not normally distributed, the Box-Cox transformation was performed [44]. The *z* score was used to evaluate the statistical significance of the coefficient of each model. All *P* values of these tests were corrected by using the Benjamini-Hochberg method [45] for multiple comparisons, and the significance level of the corrected *P* value was set to .05. All linear mixed models were implemented by using the lme4 package for R software version 3.6.1 (R Foundation for Statistical Computing).

In order to identify and compare the relationship between self-reported sleep and self-reported depression among different study sites, Spearman correlations were calculated between the PHQ-8 score and sleep subscore on the 3 study sites separately.

An example of such a 3-level linear mixed model is as follows:

*Sleep_ijk_* = *δ*_000_ + *V*_00_*_k_* + *U*_0_*_jk_* + *β*_1_(*PHQ*8*_ijk_*) + *β*_2_(*age*_jk_) + *β*_3_(*gender_jk_*) + *β*_4_(*education_jk_*) + *β*_5_(*income_jk_*) + *ε_ijk_*

where PHQ8_ijk_ is the i^th^ PHQ-8 score of the participant j of the site k, Sleep_ijk_ is one sleep feature extracted in ∆t before the i^th^ PHQ-8 record of the participant j of the site k, age_jk_, gender_jk_ , education_jk_, and income_jk_ are potential confounding variables of the participant j of the site k, *ε*_ijk_ is the residual, *δ*_000_ is the fixed effect on intercept, U_0jk_ is the random intercept of the participant j in the site k, and V_00k_ is the random intercept of the site k.

## Results

### Data Summary

**Figure 1:**
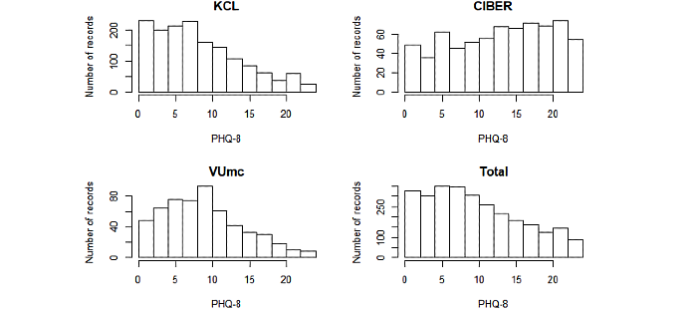
Histograms of the PHQ-8 scores of the three study sites and the entire dataset.

**Figure 2:**
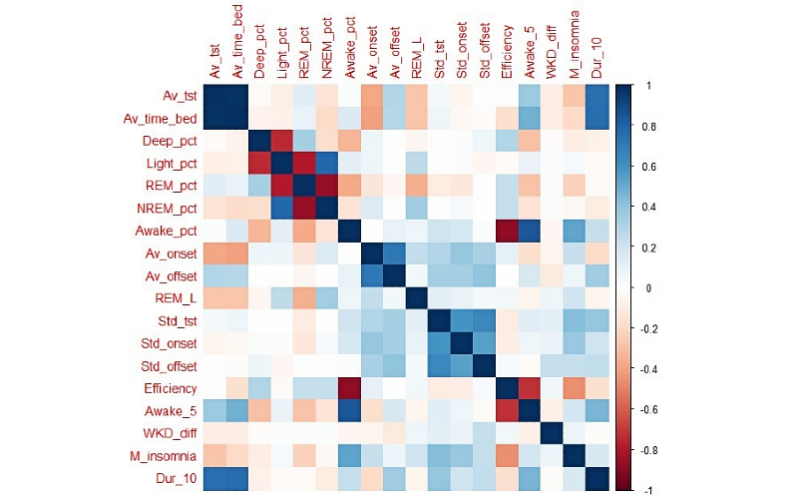
Correlation plot of pairwise Spearman correlations between all sleep features. Descriptions of abbreviations of sleep features are shown in Table 1.

According to our data inclusion criteria, from June 2018 to February 2020, 2812 PHQ-8 records from 368 participants collected from 3 study sites were included for our analysis. A summary of the sociodemographic characteristics of these participants at baseline and scores of all PHQ-8 records is shown in Table 2. The Kruskal-Wallis test was used to determine whether there were any significant differences for these characteristics between the sites. These tests revealed that, except for gender, sociodemographic characteristics and distribution of PHQ-8 scores differed between the study sites. The histograms of PHQ-8 scores of the study sites and the entire dataset are shown in Figure 1. We can observe that the KCL site had the most PHQ-8 records among the sites. PHQ-8 scores from the CIBER site were relatively high, probably because participants in the CIBER site came from a clinical population. Figure 2 presents pairwise Spearman correlation coefficients between all 18 sleep features. Table 3 shows the results of Spearman correlation analysis; we can observe there was a strong positive correlation between the sleep subscore and PHQ-8 score (*r*=.73, *z*=54.48, *P*<.001) on the entire dataset, but this correlation was relatively weaker on the VUmc data (*r*=.64, *z*=18.75, *P*<.001).

**Table 2:** A summary of sociodemographic characteristics and PHQ-8 records of participants from the 3 study sites and results of Kruskal-Wallis tests on these characteristics.

Characteristic		KCL^a^	CIBER^b^	VUmc^c^	*P* value^d^
Participants, n		189	96	83	—^e^
PHQ-8^f^ records, n		1547	708	557	—
PHQ-8 scores, median (Q1, Q3)		8 (4, 12)	14 (8, 19)	9 (5, 13)	<.001
The PHQ-8 score ≥10, n (%)		599 (38.7)	492 (69.5)	248 (44.5)	<.001
Age at baseline, median (Q1, Q3)		46 (30.3, 59.0)	55 (49.3, 60.8)	42 (28.0, 57.0)	<.001
Female sex, n (%)		144 (76.2)	69 (71.9)	65 (81.9)	.62
**Education^g^, n (%)**		—	—	—	<.001
	Degree or above	116 (61.4)	21 (21.9)	40 (48.2)	—
	Below degree	73 (38.6)	75 (78.1)	43 (51.8)	—
**Annual income^h^ (₤), n (%)**		—	—	—	.009
	<15,000	40 (21.2)	28 (29.2)	24 (28.9)	—
	15,000-40,000	80 (42.3)	53 (55.2)	34 (41.0)	—
	>40,000	67 (35.5)	15 (15.6)	14 (16.9)	—
	Not mentioned	2 (1.1)	0 (0)	11 (13.3)	—

^a^KCL: King’s College London.

^b^CIBER: Centro de Investigación Biomédican en Red.

^c^VUmc: Vrije Universiteit Medisch Centrum.

^d^*P* value of Kruskal-Wallis test.

^e^Not applicable.

^f^PHQ-8: Patient Health Questionnaire 8-item.

^g^Education levels of Spain and the Netherlands transformed into equivalent British education levels.

^h^Annual income levels of Spain and the Netherlands transformed into equivalent British levels.

**Table 3:** Spearman correlation coefficients between the PHQ-8 score and sleep subscore^a^ on the 3 study sites and their 95% confidence intervals, z score statistics, and *P* values.

Study site	*r*	95% CI	*z* score	*P* value
KCL^b^	.74	0.71, 0.76	41.99	<.001
CIBER^c^	.78	0.75, 0.81	32.09	<.001
VUmc^d^	.64	0.58, 0.69	18.75	<.001
Total	.73	0.71, 0.74	54.48	<.001

^a^Sleep subscore represents the score of subitem 3 in the PHQ-8.

^b^KCL: King’s College London.

^c^CIBER: Centro de Investigación Biomédican en Red.

^d^VUmc: Vrije Universiteit Medisch Centrum.

### Three-Level Linear Mixed Models on the Entire Dataset

Table 4 shows the results from 3-level linear mixed regression models that reflect the associations between sleep features and the PHQ-8 score and sleep subscore, respectively. A total of 14 sleep features were found to be significantly associated with the PHQ-8 score, among them awake percentage (*z*=5.45, *P*<.001), awakening times (*z*=5.53, *P*<.001), insomnia (*z*=4.55, *P*<.001), mean sleep offset time (*z*=6.19, *P*<.001), and hypersomnia (*z*=5.30, *P*<.001) were the top 5 features ranked by *z* score statistics. The percentages of light sleep (*Light_pct*) and NREM sleep (*NREM_pct*) and sleep efficiency (*Efficiency*) were significantly and negatively associated with the PHQ-8 score, whereas the rest of the significant features were positively associated with the PHQ-8 score.

**Table 4:** Slope coefficient estimates, 95% confidence intervals, z score statistics, and *P* values from 3-level linear mixed models on the entire dataset for exploring associations between sleep features^a^ and the PHQ-8 score and sleep subscore^b^.

Features	PHQ-8^c^ score					Sleep subscore				
	Coeff.^d^	95% CI	z score	*P* value	Coeff.		95% CI	z score	*P* value	
Av_tst	0.013	0.006, 0.019	3.93	<.001	–0.004		–0.034, 0.025	–0.28	.78	
Av_time_bed	0.016	0.009, 0.023	4.45	<.001	0.005		–0.028, 0.038	0.29	.77	
Deep_pct	–0.007	–0.026, 0.011	–0.75	.45	–0.104		–0.191, –0.017	–2.34	.02	
Light_pct	–0.032	–0.064, –0.001	–2.02	.04	0.090		–0.057, 0.237	1.20	.23	
REM_pct	0.003	–0.021, 0.027	0.25	.80	–0.125		–0.238, –0.012	–2.17	.03	
NREM_pct	–0.038	–0.062, –0.014	–3.12	.002	–0.014		–0.127, 0.098	–0.25	.80	
Awake_pct	0.035	0.022, 0.048	5.45	<.001	0.139		0.079, 0.199	4.58	<.001	
Av_onset	0.007	–0.001, 0.015	1.71	.09	0.078		0.040, 0.115	4.03	<.001	
Av_offset	0.025	0.017, 0.033	6.19	<.001	0.097		0.060, 0.135	5.10	<.001	
REM_L	0.034	–0.021, 0.088	1.21	.23	0.085		–0.178, 0.347	0.63	.53	
Std_tst	0.008	0.004, 0.012	4.07	<.001	0.047		0.028, 0.067	4.77	<.001	
Std_onset	0.012	0.004, 0.019	3.11	.002	0.060		0.022, 0.097	3.13	.002	
Std_offset	0.012	0.005, 0.018	3.58	<.001	0.069		0.037, 0.100	4.26	<.001	
Efficiency	–0.025	–0.037, –0.012	–3.91	<.001	–0.108		–0.167, –0.050	–3.65	<.001	
Awake_5	0.016	0.010, 0.022	5.53	<.001	0.038		0.011, 0.065	2.77	.006	
WKD_diff	0.134	0.039, 0.230	2.76	.006	0.747		0.255, 1.240	2.98	.003	
M_insomnia	0.370	0.211, 0.530	4.55	<.001	2.373		1.595, 3.151	5.98	<.001	
Dur_10	0.309	0.195, 0.423	5.30	<.001	0.909		0.357, 1.462	3.23	.001	

^a^Definitions of sleep features in this table are shown in Table 1.

^b^Sleep subscore represents the score of subitem 3 in the PHQ-8.

^c^PHQ-8: Patient Health Questionnaire 8-item.

^d^Slope coefficient estimates for all sleep features.

For sleep subscore, we can notice that deep sleep percentage (*Deep_pct*), REM sleep percentage (*REM_pct*), and sleep efficiency (*Efficiency*) were significantly and negatively associated with the sleep subscore, whereas features of the percentage of awake time (*Awake_pct*), unstable sleep (*Std_tst*, *Std_onset*, *Std_offset*), awakening times (*Awake_5*), weekend catch-up sleep (*WKD_diff*), sleep onset time (*Av_onset*), sleep offset time (*Av_offset*), insomnia (*M_insomnia*), and hypersomnia (*Dur_10*) were significantly and positively associated with the sleep subscore.

### Two-Level Linear Mixed Models on Different Research Sites

Table 5 provides the results from 2-level linear mixed models which show the associations between sleep features and the PHQ-8 score on different research sites separately. On the KCL data, most associations between sleep features and depression were consistent with the results on the entire dataset. On the CIBER data, some features were no longer significantly associated with the PHQ-8 score. However, on the VUmc data, most features lost their significance except features of total sleep time (*Av_tst*), time in bed (*Av_time_bed*), REM latency (*REM_L*), and awakenings (*Awake_5*).

Table 6 shows associations between sleep features and the sleep subscore on different research sites. The significance of associations between sleep features and the sleep subscore were different among the 3 study sites. Notably, the insomnia feature (*M_insomnia*) and at least one feature of sleep stability were significantly positively associated with sleep subscore on the data of all 3 sites.

**Table 5:** Coefficient estimates, 95% confidence intervals, and *P* values from 2-level linear mixed models on the 3 study sites for exploring associations between sleep features^a^ and the PHQ-8 score.

Features	KCL^b^				CIBER^c^				VUmc^d^		
	Coeff.^e^	95% CI	*P* value	Coeff.		95% CI	*P* value	Coeff.		95% CI	*P* value
Av_tst	0.013	0.005, 0.020	.001	0.016		–0.001, 0.033	.06	0.011		0, 0.022	.049
Av_time_bed	0.016	0.008, 0.024	<.001	0.021		0.002, 0.040	.03	0.013		0.001, 0.025	.04
Deep_pct	–0.005	–0.028, 0.018	.69	0.024		–0.022, 0.071	.31	–0.037		–0.074, 0.001	.06
Light_pct	–0.046	–0.087, –0.006	.03	–0.081		–0.155, –0.007	.03	0.019		–0.043, 0.082	.55
REM_pct	0.013	–0.018, 0.043	.43	0.015		–0.042, 0.071	.62	–0.007		–0.055, 0.041	.77
NREM_pct	–0.049	–0.080, –0.018	.002	–0.060		–0.116, –0.005	.04	–0.016		–0.062, 0.030	.50
Awake_pct	0.037	0.020, 0.054	<.001	0.043		0.015, 0.071	.003	0.022		–0.003, 0.047	.09
Av_onset	0.010	0.000, 0.020	.047	0.004		–0.018, 0.025	.74	–0.005		–0.021, 0.010	.52
Av_offset	0.029	0.018, 0.039	<.001	0.024		0.004, 0.043	.02	0.012		–0.004, 0.029	.14
REM_L	0.019	–0.049, 0.088	.58	0.106		–0.026, 0.237	.12	–0.126		–0.231, –0.020	.02
Std_tst	0.008	0.003, 0.013	.001	0.009		0, 0.019	.06	0.002		–0.006, 0.010	.62
Std_onset	0.007	–0.002, 0.016	.14	0.019		–0.001, 0.039	.06	0.001		–0.011, 0.013	.93
Std_offset	0.009	0.001, 0.017	.03	0.019		0.002, 0.036	.03	0.003		–0.008, 0.015	.56
Efficiency	–0.025	–0.041, –0.008	.004	–0.043		–0.071, –0.016	.002	–0.012		–0.037, 0.013	.34
Awake_5	0.014	0.006, 0.022	<.001	0.022		0.009, 0.035	.001	0.016		0.005, 0.027	.01
WKD_diff	0.211	0.084, 0.339	.001	0.071		–0.126, 0.268	.48	0.077		–0.144, 0.299	.49
M_insomnia	0.472	0.259, 0.685	<.001	0.381		0.028, 0.734	.04	–0.048		–0.385, 0.289	.78
Dur_10	0.331	0.191, 0.472	<.001	0.340		0.052, 0.627	.02	0.181		–0.051, 0.413	.13

^a^Definitions of sleep features in this table are shown in Table 1.

^b^KCL: King’s College London.

^c^CIBER: Centro de Investigación Biomédican en Red.

^d^VUmc: Vrije Universiteit Medisch Centrum.

^e^Slope coefficient estimates for all sleep features.

**Table 6:** Coefficient estimates, 95% confidence intervals, and *P* values from 2-level linear mixed models on the 3 study sites for exploring associations between sleep features^a^ and the sleep subscore^b^.

Features	KCL^c^				CIBER^d^				VUmc^e^		
	Coeff.^f^	95% CI	*P* value	Coeff.		95% CI	*P* value	Coeff.		95% CI	*P* value
Av_tst	0.015	–0.021, 0.050	.41	–0.035		–0.116, 0.047	.41	–0.017		–0.070, 0.035	.52
Av_time_bed	0.026	–0.013, 0.066	.19	–0.025		–0.116, 0.065	.58	–0.015		–0.074, 0.043	.61
Deep_pct	–0.027	–0.134, 0.081	.63	–0.196		–0.412, 0.020	.07	–0.191		–0.369, –0.014	.04
Light_pct	–0.024	–0.213, 0.166	.81	0.098		–0.250, 0.445	.58	0.312		0.016, 0.608	.04
REM_pct	–0.116	–0.260, 0.028	.12	–0.037		–0.304, 0.230	.79	–0.169		–0.398, 0.060	.15
NREM_pct	–0.048	–0.194, 0.098	.52	–0.123		–0.389, 0.143	.37	0.125		–0.096, 0.346	.27
Awake_pct	0.165	0.085, 0.245	<.001	0.150		0.020, 0.280	.02	0.049		–0.073, 0.170	.43
Av_onset	0.055	0.008, 0.101	.02	0.075		–0.023, 0.172	.13	0.128		0.054, 0.202	.001
Av_offset	0.102	0.053, 0.150	<.001	0.048		–0.040, 0.135	.29	0.133		0.056, 0.210	.001
REM_L	0.073	–0.255, 0.401	.66	0.146		–0.494, 0.787	.65	–0.171		–0.683, 0.340	.51
Std_tst	0.046	0.022, 0.071	<.001	0.046		–0.002, 0.094	.06	0.043		0.004, 0.082	.03
Std_onset	0.028	–0.015, 0.070	.21	0.089		–0.018, 0.195	.10	0.079		0.020, 0.139	.01
Std_offset	0.046	0.008, 0.084	.02	0.109		0.022, 0.195	.01	0.072		0.016, 0.127	.01
Efficiency	–0.118	–0.196, –0.041	.003	–0.152		–0.280, –0.024	.02	–0.044		–0.162, 0.074	.46
Awake_5	0.047	0.011, 0.083	.01	0.037		–0.022, 0.097	.22	0.013		–0.042, 0.067	.65
WKD_diff	1.169	0.534, 1.804	<.001	0.210		–0.864, 1.284	.70	0.283		–0.830, 1.395	.62
M_insomnia	2.302	1.274, 3.329	<.001	2.777		1.070, 4.485	.001	1.823		0.180, 3.465	.03
Dur_10	1.057	0.387, 1.728	.002	0.576		–0.844, 1.995	.43	0.706		–0.411, 1.823	.22

^a^The definitions of sleep features in this table are shown in Table 1.

^b^The sleep subscore represents the score of subitem 3 in the PHQ-8.

^c^KCL: King’s College London.

^d^CIBER: Centro de Investigación Biomédican en Red.

^e^VUmc: Vrije Universiteit Medisch Centrum.

^f^Slope coefficient estimates for all sleep features.

## Discussion

### Principal Findings

In this study, we extracted 18 sleep features through Fitbit data to quantitatively describe participant sleep characteristics in 5 categories (sleep architecture, sleep stability, sleep quality, insomnia, and hypersomnia) associated with the severity of depression. Along with the depressive status worsening, the following changes may be seen in the past 2 weeks: (1) percentage of light/NREM sleep decreased and the percentage of wakefulness during sleep increased (sleep architecture); (2) sleep duration/onset/offset were unstable (sleep stability); (3) reduced sleep efficiency, more awakenings during sleep, and longer weekend catch-up sleep were observed (sleep quality); (4) more days with insomnia were observed (insomnia); (5) more days with hypersomnia were observed (hypersomnia). Table 4 illustrated that our sleep features of these 5 categories could reflect both the participant sleep condition (sleep subscore) and depressive symptom severity (PHQ-8 score) of the past 2 weeks.

### Potential Factors Affecting Associations

We evaluated our models on the research sites separately. From Table 5 and Table 6, we can notice that the associations between sleep features and PHQ-8 score/sleep subscore varied across different sites. Several factors may affect the associations. First, the populations of the 3 sites were significantly different (Table 2). For example, participants in the CIBER site came from a clinical population and their average age was oldest, so one speculation is that there was less difference between their weekday sleep and weekend sleep for inpatients or people in retirement. Therefore, this may be the reason why the feature of weekend catch-up sleep (*WKD_diff*) lost significance on the CIBER data. In addition, the reduced significance of features related to sleep onset and offset time on the CIBER site might be related to the regular sleep pattern in CIBER site favors going to bed later, as seen in our previous study [42].

The associations between sleep features and the sleep subscore on the VUmc data (Table 6) were similar to that in the entire dataset (Table 4), which demonstrated sleep features have the same ability to capture the sleep condition of participants on the VUmc data. However, the significance of associations between these sleep features and the PHQ-8 score was reduced in the VUmc data (Table 5). One possible reason is that, as seen on Table 3, the correlation between the sleep subscore and PHQ-8 score in the VUmc data (*r*=.64) was weaker than other 2 study sites (KCL: *r*=.74 and CIBER: *r*=.78), which may be caused by confounding variables that we did not consider or record in the VUmc population such as medication and occupational status.

Sample size and heterogeneity of the dataset were other possible factors that may affect results. Table 2 shows that the KCL site had the most PHQ-8 records, whereas VUmc had the least data. As depression manifests itself in distinctive symptoms on different people, it may be difficult to fully explore the associations between sleep and depression on a relatively smaller dataset (VUmc). For example, hypersomnia is specifically related to bipolar patients [7,8]; therefore, if the dataset did not contain enough bipolar patients or bipolar patients were not in depressive episodes when they completed their PHQ-8 records, it would be hard to find the association between hypersomnia and depression.

### Comparison With Prior Work

**Figure 3:**
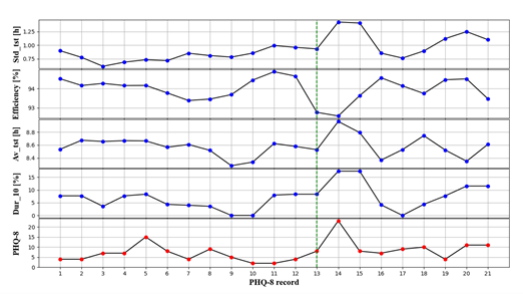
The PHQ-8 scores and a select 4 sleep features of one participant with an obvious increasing trend in PHQ-8 score at 13th PHQ-8 record. Descriptions of abbreviations of sleep features in this figure are shown in Table 1.

Our study has a relatively larger sample size and a longer follow-up duration than previous studies on monitoring depression by using wearable devices and mobile phones [19-21]. Each participant has multiple PHQ-8 records and repeated measurements of sleep, so we can not only explore the relationships between sleep and depression between individuals but also find the associations within individuals by using the linear mixed model. Figure 3 is an example of a possible depression relapse of one participant, showing an obvious increasing trend in PHQ-8 scores at the 13th PHQ-8 record of this participant. We can observe the sleep features in Figure 3 are significantly associated with the PHQ-8 score. This indicates that the sleep features extracted in this paper have the potential to be the biomarkers of depression.

We also compared our findings with previous studies that used other measurements to assess sleep, such as PSG and sleep questionnaires. Although the sample size, population, measurements, duration of these studies are different, the comparison may help to find more general associations between sleep and depression. Table 7 provides a summary of the comparison. Several longitudinal studies based on sleep questionnaires have shown that insomnia and hypersomnia are both symptoms of depression [6,46], which we found in our research. Kang et al [36] found the weekend catch-up sleep was significantly positively correlated with the severity of depression by analyzing the self-sleep questionnaires of 4553 Korean adolescents, and this is consistent with the finding in our paper. A sleep report has shown that higher sleep efficiency, more deep sleep, and fewer awakenings after sleep onset represent better sleep quality [32], which is also consistent with the relationships we found between deep sleep percentage, awake percentage, and awakenings (>5 minutes) with sleep subscore. A review showed that according to PSG research, the shortened REM latency and increased percentage of REM sleep are biological markers of depression relapse [9]; however, relationships between depressive symptom severity with REM sleep percentage and REM latency were not significant in our results.

**Table 7:** Summary of the comparisons with previous studies using other measurements to assess sleep.

Type of feature	Findings in previous studies	Consistent^a^	Measurement
Insomnia	Insomnia is significantly related to depression [6].	Yes	Questionnaire
Hypersomnia	Prevalence of hypersomnia is high in depressed patients [46].	Yes	Questionnaire
Weekend catch-up sleep	Weekend catch-up sleep is significantly positively correlated with the severity of depression [36].	Yes	Questionnaire
Deep sleep percentage	More deep sleep represents higher sleep quality [32].	Yes	Questionnaire
Awake percentage, Awakenings (>5 mins)	Fewer awakenings after sleep onset represents better sleep quality [32].	Yes	Questionnaire
Sleep efficiency	Higher sleep efficiency represents better sleep quality [32].	Yes	Questionnaire
REM sleep percentage	Increased REM sleep percentage can be biomarkers of depression [9].	No	Polysomnography
REM^b^ latency	Shortened REM latency can be biomarkers of depression [9].	No	Polysomnography

^a^Whether it is consistent with our findings.

^b^REM: rapid eye movement.

### Limitations

Missing data is the major hindrance in our study. For various reasons, there were many missing records of sleep. We set the completion rate of sleep records greater than 85% (12 days) as one of the data inclusion criteria. However, the optimum threshold is unclear, which needs to be further studied in future research. Missingness could also be associated with depressive status and could be a useful marker of relapse of depression; for example, participants may not feel like complying if they are feeling depressed. In future research, we will consider missingness as a potential feature.

Although we adjusted our models for age, gender, education level, and annual income, it is hard to consider all potential confounding variables. For example, some participants with sleep disorders may take sleep medications. Sleep medications have a significant influence on the features of sleep. Unfortunately, there was no daily record of whether the participant took medication. This confounding variable may affect the result.

The data of sleep stages used in this paper were provided by the Fitbit wristband. According to their validation studies, the Fitbit wristband showed promise in detecting sleep-wake states but limitations in other sleep stages estimation [27-29]. This may be the reason the features of REM percentage and REM latency in our paper did not show significant relationships with depressive symptoms. For detecting insomnia, the sleep onset latency (SOL) in the PSG report is a reliable indicator of insomnia, but the Charge 2 and 3 are not able to measure SOL directly. The features related to insomnia in our paper can partially reflect insomnia, but they may be affected by factors (such as work schedules or activities) other than insomnia. Therefore, in future research, we will combine multiple features (such as a late sleep onset time accompanied by a short total sleep time) to determine whether a participant has insomnia and try to use activity information (eg, steps) provided by Fitbit to approximate SOL. Although there are some limitations of Fitbit data, it provides a means to investigate sleep characteristic in home settings.

In feature extraction, we did not consider the impact of individual circumstances on sleep features. For example, some participants may need to shift work at night, which our features are unable to capture. We will consider the impact of sleep habits and lifestyles on sleep features in the future. Further, we did not explore the impact of individual patterns of depression [47]—for example, the distinction between people with typical and atypical depression who report reduced and increased sleep, respectively, during depressive episodes. In future work, we will explore whether including this dimension improves specificity of our findings.

In this paper, we focused on analyzing the manifestations of depression in sleep characteristics. We will investigate whether these relationships are bidirectional in future research. We only performed bivariate analysis (ie, separately analyzing the association between each feature and the PHQ-8 score). The combination of features and nonlinear relationships was not considered. We will try to apply machine/deep learning models to predict the severity of depression by using sleep features in future research.

### Conclusions

Although consumer wearable devices may not be a substitute for PSG to assess sleep quality accurately, we demonstrated that some derived sleep features extracted from these wearable devices show potential for remote measurement of sleep and consequently can act as a biomarker of depression in real-world settings. These findings may provide the basis for the development of clinical tools that could be used to passively monitor disease state and trajectory with minimal burden on the participant.

## Abbreviations

CIBER: Centro de Investigación Biomédican en Red
EFPIA: European Federation of Pharmaceutical Industries and Associations
KCL: King’s College London
NHS: National Health Service
NIHR: National Institute for Health Research
NREM: non-REM
PHQ-8: Patient Health Questionnaire 8-item
PSG: polysomnography
PSQI: Pittsburgh Sleep Quality Index
RADAR-CNS: Remote Assessment of Disease and Relapse–Central Nervous System
RADAR-MDD: Remote Assessment of Disease and Relapse–Major Depressive Disorder
REM: rapid eye movement
SOL: sleep onset latency
VUmc: Vrije Universiteit Medisch Centrum
WHO: World Health Organization

## References

[1] (2017). Depression and other common mental disorders: global health estimates.

[2] Cuijpers P, Schoevers RA (2004). Increased mortality in depressive disorders: a review. Curr Psychiatry Rep.

[3] Lenox-Smith A, Macdonald MTB, Reed C, Tylee A, Peveler R, Quail D, Wildgust HJ (2013). Quality of life in depressed patients in UK primary care: the FINDER study. Neurol Ther.

[4] Lerner D, Adler DA, Chang H, Berndt ER, Irish JT, Lapitsky L, Hood MY, Reed J, Rogers WH (2004). The clinical and occupational correlates of work productivity loss among employed patients with depression. J Occup Environ Med.

[5] Mendelson WB (2012). Human Sleep and Its Disorders.

[6] Alvaro PK, Roberts RM, Harris JK (2013). A systematic review assessing bidirectionality between sleep disturbances, anxiety, and depression. Sleep.

[7] Detre T, Himmelhoch J, Swartzburg M, Anderson CM, Byck R, Kupfer DJ (1972). Hypersomnia and manic-depressive disease. Am J Psychiatry.

[8] Thase ME, Himmelhoch JM, Mallinger AG, Jarrett DB, Kupfer DJ (1989). Sleep EEG and DST findings in anergic bipolar depression. Am J Psychiatry.

[9] Palagini L, Baglioni C, Ciapparelli A, Gemignani A, Riemann D (2013). REM sleep dysregulation in depression: state of the art. Sleep Med Rev.

[10] Riemann D, Berger M, Voderholzer U (2001). Sleep and depression—results from psychobiological studies: an overview. Biol Psychol.

[11] Iber C, Ancoli-Israel S, Chesson A, Quan S (2007). AASM Manual for the Scoring of Sleep and Associated Events.

[12] Sánchez-Ortuño MM, Edinger JD, Means MK, Almirall D (2010). Home is where sleep is: an ecological approach to test the validity of actigraphy for the assessment of insomnia. J Clin Sleep Med.

[13] Buysse DJ, Reynolds CF, Monk TH, Berman SR, Kupfer DJ (1989). The Pittsburgh Sleep Quality Index: a new instrument for psychiatric practice and research. Psychiatry Res.

[14] Moore CM, Schmiege SJ, Matthews EE (2015). Actigraphy and sleep diary measurements in breast cancer survivors: discrepancy in selected sleep parameters. Behav Sleep Med.

[15] Beattie Z, Oyang Y, Statan A, Ghoreyshi A, Pantelopoulos A, Russell A, Heneghan C (2017). Estimation of sleep stages in a healthy adult population from optical plethysmography and accelerometer signals. Physiol Meas.

[16] Van de Water ATM, Holmes A, Hurley DA (2011). Objective measurements of sleep for non-laboratory settings as alternatives to polysomnography—a systematic review. J Sleep Res.

[17] Zhang Y, Yang Z, Lan K (2019). Sleep stage classification using bidirectional lstm in wearable multi-sensor systems.

[18] Zhang Y, Yang Z, Zhang Z (2018). Breathing disorder detection using wearable electrocardiogram and oxygen saturation. Proc 16th ACM Conf Emb Netw Sensor Syst.

[19] Miwa H, Sasahara S, Matsui T (2007). Roll-over detection and sleep quality measurement using a wearable sensor. Annu Int Conf IEEE Eng Med Biol Soc.

[20] Mark G, Czerwinski M, Iqbal S (2016). Workplace indicators of mood: behavioral and cognitive correlates of mood among information workers. Proc 6th Int Conf on Dig Health.

[21] Demasi O, Aguilera A, Recht B (2016). Detecting change in depressive symptoms from daily wellbeing questions, personality, and activity. IEEE.

[22] Khoulji S, Garzón-Rey J, Aguilo J (2017). Remote Assessment of Disease and Relapse–Central Nervous System (RADAR-CNS). Transact Mach Learn Artif Intell.

[23] Matcham F, Barattieri di San Pietro C, Bulgari V, de Girolamo G, Dobson R, Eriksson H, Folarin AA, Haro JM, Kerz M, Lamers F, Li Q, Manyakov NV, Mohr DC, Myin-Germeys I, Narayan V, Bwjh P, Ranjan Y, Rashid Z, Rintala A, Siddi S, Simblett SK, Wykes T, Hotopf M, RADAR-CNS consortium (2019). Remote assessment of disease and relapse in major depressive disorder (RADAR-MDD): a multi-centre prospective cohort study protocol. BMC Psychiatry.

[24] Hersenonderzoek.nl.

[25] Ranjan Y, Rashid Z, Stewart C, Conde P, Begale M, Verbeeck D, Boettcher S, Dobson R, Folarin A, RADAR-CNS Consortium (2019). RADAR-Base: open source mobile health platform for collecting, monitoring, and analyzing data using sensors, wearables, and mobile devices. JMIR Mhealth Uhealth.

[26] Kroenke K, Strine TW, Spitzer RL, Williams JBW, Berry JT, Mokdad AH (2009). The PHQ-8 as a measure of current depression in the general population. J Affect Disord.

[27] de Zambotti M, Goldstone A, Claudatos S, Colrain IM, Baker FC (2018). A validation study of Fitbit Charge 2 compared with polysomnography in adults. Chronobiol Int.

[28] Haghayegh S, Khoshnevis S, Smolensky MH, Diller KR, Castriotta RJ (2019). Accuracy of wristband fitbit models in assessing sleep: systematic review and meta-analysis. J Med Internet Res.

[29] Liang Z, Chapa-Martell MA (2019). Accuracy of fitbit wristbands in measuring sleep stage transitions and the effect of user-specific factors. JMIR Mhealth Uhealth.

[30] Aluoja A, Leinsalu M, Shlik J, Vasar V, Luuk K (2004). Symptoms of depression in the Estonian population: prevalence, sociodemographic correlates and social adjustment. J Affect Disord.

[31] Akhtar-Danesh N, Landeen J (2007). Relation between depression and sociodemographic factors. Int J Ment Health Syst.

[32] Ohayon M, Wickwire EM, Hirshkowitz M, Albert SM, Avidan A, Daly FJ, Dauvilliers Y, Ferri R, Fung C, Gozal D, Hazen N, Krystal A, Lichstein K, Mallampalli M, Plazzi G, Rawding R, Scheer FA, Somers V, Vitiello MV (2017). National Sleep Foundation's sleep quality recommendations: first report. Sleep Health.

[33] Walker MP (2008). Sleep-dependent memory processing. Harv Rev Psychiatry.

[34] Rasch B, Born J (2013). About sleep's role in memory. Physiol Rev.

[35] Manni R (2005). Rapid eye movement sleep, non-rapid eye movement sleep, dreams, and hallucinations. Curr Psychiatry Rep.

[36] Kang S, Lee YJ, Kim SJ, Lim W, Lee H, Park Y, Cho IH, Cho S, Hong JP (2014). Weekend catch-up sleep is independently associated with suicide attempts and self-injury in Korean adolescents. Compr Psychiatry.

[37] Liu X, Zhao Z, Jia C, Buysse DJ (2008). Sleep patterns and problems among chinese adolescents. Pediatrics.

[38] American Psychiatric Association (2013). Diagnostic and Statistical Manual of Mental Disorders (DSM-5).

[39] Tam EM, Lam RW, Robertson HA, Stewart JN, Yatham LN, Zis AP (1997). Atypical depressive symptoms in seasonal and non-seasonal mood disorders. J Affect Disord.

[40] Farhan AA, Yue C, Morillo R (2016). Behavior vs. introspection: refining prediction of clinical depression via smartphone sensing data.

[41] Singer JD, Willett JB, Willett JB (2003). Applied Longitudinal Data Analysis: Modeling Change and Event Occurrence.

[42] Sun S, Folarin AA, Ranjan Y, Rashid Z, Conde P, Stewart C, Cummins N, Matcham F, Dalla Costa G, Simblett S, Leocani L, Lamers F, Sørensen PS, Buron M, Zabalza A, Guerrero Pérez AI, Penninx BW, Siddi S, Haro JM, Myin-Germeys I, Rintala A, Wykes T, Narayan VA, Comi G, Hotopf M, Dobson RJ, RADAR-CNS Consortium (2020). Using smartphones and wearable devices to monitor behavioral changes during COVID-19. J Med Internet Res.

[43] Laird NM, Ware JH (1982). Random-effects models for longitudinal data. Biometrics.

[44] Box GEP, Cox DR (2018). An analysis of transformations. J Royal Stat Soc B.

[45] Benjamini Y, Hochberg Y (2018). Controlling the false discovery rate: a practical and powerful approach to multiple testing. J Royal Stat Soc B.

[46] Kaplan KA, Harvey AG (2009). Hypersomnia across mood disorders: a review and synthesis. Sleep Med Rev.

[47] Brailean A, Curtis J, Davis K, Dregan A, Hotopf M (2019). Characteristics, comorbidities, and correlates of atypical depression: evidence from the UK Biobank Mental Health Survey. Psychol Med.

